# The Nervous System Contributes to the Tumorigenesis and Progression of Human Digestive Tract Cancer

**DOI:** 10.1155/2022/9595704

**Published:** 2022-03-07

**Authors:** Dayou Dai, Hao Liu

**Affiliations:** ^1^Second Clinical Medical College, Southern Medical University, Guangzhou, 510515, China; ^2^Department of General Surgery, Nanfang Hospital, Southern Medical University, Guangzhou, Guangdong 510515, China

## Abstract

Tumors of the gastrointestinal tract are one of the highest incidences of morbidity and mortality in humans. Recently, a growing number of researchers have indicated that nerve fibers and nerve signals participate in tumorigenesis. The current overarching view based on the responses to therapy revealed that tumors are partly promoted by the tumor microenvironment (TME), endogenous oncogenic factors, and complex systemic processes. Homeostasis of the neuroendocrine-immune axis (NEI axis) maintains a healthy in vivo environment in humans, and dysfunction of the axis contributes to various cancers, including the digestive tract. Interestingly, nerves might promote tumor development via multiple mechanisms, including perineural invasion (PNI), central level regulation, NEI axis effect, and neurotransmitter induction. This review focuses on the association between digestive tumors and nerve regulation, including PNI, the NEI axis, stress, and neurotransmitters, as well as on the potential clinical application of neurotherapy, aiming to provide a new perspective on the management of digestive cancers.

## 1. Introduction

Currently, digestive cancer is a multifactorial, dangerous, and complex disease. The denervation treatment for PDAC and gastric tumors suggests that there is a connection between nerve fibers and cancer tissues. Nerves exert multiple effects on the TME, and PNI also participates in gastrointestinal tumorigenesis. Studies are focusing on the connection of nerves and cancer. In recent decades, neurotransmitters have been studied to provide a new strategy to treat tumors. This paper is aimed at reviewing the connection between nerves and cancers in the digestive system.

## 2. Peripheral Neural and Central

### 2.1. Peripheral Neural: Nerve Cells Promote Gastrointestinal Tumors by PNI

PNI, a process of tumors invading nerves, is now a popular research prospect. It has been suggested that PNI promotes the mutual effect between tumor cells and nerve fibers, which may lead to unpredicted tumorigenesis [1]. Exciting achievements made by many researchers show that PNI is gradually emerging as a key pathological feature of gastrointestinal malignancies, such as tumors of the pancreas, colon, rectum, biliary tract, and stomach [2]. PNI contributes to tumorigenesis via multiple pathways involved in almost all aspects of NEI homeostasis, either of immune or hormonal function. Meanwhile, removing the peripheral nerves (PNSs) located in various sites prevented cancer development in several preclinical models [3], which is regarded as compelling evidence of cancer nerve dependence. Abnormal changes, including changes in size, density, and number, in the nervous system are closely associated with tumors [4]. According to these studies, PNI of sensory and autonomic nerves plays one of the most important roles in gastrointestinal tumor development and is discussed worldwide.

In sensory nerve pathways, a reciprocal signaling loop was also found between the pancreas and sensory neurons. This interaction was regarded as evidence of the participation of inflammation in Kras-concerned neoplasia [3]. Neurotransmitters produced in many different organs and nerve endings trigger the development of cancer and promote tumor metastasis. These effects are believed to be achieved by stimulating the corresponding receptors on the membrane, resulting in the release of neurotrophic growth factors, which promote nerve growth and accelerate their invasion in pancreatic tumors [5]. In this way, nerves and tumors can interact with each other.

Tumor cell growth requires the invasion of nerve fibers, a process that is similar to regeneration [5]. The neoneurogenesis of tumors is like either neoangiogenesis or lymph angiogenesis. The formation of new nerves in tumor tissue is an important step in promoting tumor progression, which is thought to be similar to the process of geckos growing a new tail after cutting off its tail [6, 7]. However, much more evidence concerning the derivation of invasive nerves in tumor tissues, the involvement of nerve signals within tumor tissues, and their biological functions is still underdeveloped [8].

### 2.2. Central Neural: Stress Exerts a Great Effect on Tumorigenesis

*(1) Negative Stress and Gastrointestinal Cancer*. In the past several decades, stress has been proven to increase the risk of tumorigenesis and is also a strong factor in the gastrointestinal system. An epidemiologic study in 281,290 individual participants found that work-related stress dramatically increased the incidence rate of colorectal cancers [9]. Activated *β*2-adrenergic receptor signaling, nerve growth factor secretion, and upregulation of catecholamines drive chronic neuropsychological stress, and this progression promotes Kras-induced pancreatic tumorigenesis. In addition to *β*2-adrenergic receptor signaling, nerve growth factor and catecholamines also facilitate chronic neuropsychological stress, thus enhancing the development of Kras-induced pancreatic tumors. In 2018, tropomyosin receptor kinase inhibitors combined with gemcitabine were once used to treat a pancreatic cancer animal model and achieved remarkable success. However, the mechanism of this effective combination therapy has not been proven [10]. In addition to promoting tumorigenesis, mental stress was also proven to exacerbate drug resistance and decrease both cellular immunity and immunosurveillance, thus aggravating tumor development [11]. Chronic stress not only inhibits cellular immune function but also inhibits cellular immune surveillance. Recent experiments have proven that either stress-induced glucocorticoid surges or TSC22D3 upregulation expressed on dendritic cells causes the body to disrupt chemotherapy or antitumor immunotherapy. In cancer patients, there is a close correlation among TSC22D3 expressions in circulating leukocytes [12].

*(2) Positive Stress Response and Cancer*. However, stress stimulation and positive factors can effectively inhibit the growth of tumors. Environmental enrichment (EE) is a traditional technical term designed to enhance sensory, cognitive, and social stimulation, and other terms are also considered necessary for the animal's optimal mental and physical health. According to the report, mice that lived in an enriched environment had less tumor growth and higher rates of colorectal cancer remission. This phenomenon was consistent with an increase in serum brain-derived neurotrophic factor (BDNF) and a significant decrease in leptin concentration [13]. Interestingly, in pancreatic cancer, through the downregulation of mitochondria-related gene expression, EE significantly reduces the weight of subcutaneous and orthotopic tumors in mouse models [14]. In terms of immune mechanisms, EE regulation and immunoenhancement in surveillance and defense play an indispensable role in anticancer events. EEs can increase the proportion of CD8+ cytotoxic T lymphocytes (CTLs) and play a defensive role. Both propranolol and mifepristone block EE-related CTL regulation, indicating that both the SNS and HPA axes are involved [15]. In most cases, natural killer (NK) cells are the main force of EE exposure-induced tumor suppression [16–18]. In addition, it induces microglia/macrophage (M/M*φ*) activation under EE conditions, suggesting that EE also plays a special immunomodulatory role in cancer. It is exciting to note that EEs have recently been reported to have an additional effect on tumor development in conjunction with immune checkpoint therapy [19]. With the effect of EE, nerves can regulate the tumor by neurotransmitters and cell factors.

The more optimistic the person is, the lower the cancer-related death rate. In Kim et al.'s report, optimists had a 16 percent lower hazard ratio for all cancers than pessimists [20]. On the other hand, physical activity in leisure time is related to multiple inhibiting effects on cancer, such as activating the sympathetic system, suppressing endocrine factors (such as gonadal hormone, insulin, and adenosine kinase), and improving immune system function [21]. As a result, NEI regulation coupled with exercise is a new prospect in cancer treatment. Environmental enrichment was originally a concept in husbandry, and in the 1940s, with the development of medical research, it gradually meant that irritating objects were placed in someone's environment to promote broader brain development. Generally, environmental enrichment-induced immune defense, immune regulation, and the enhancement of immune surveillance play a strong role in anticancer events.

## 3. Neurosignal and Gastrointestinal Tumor

Many neurosignals, such as cytokines, transmitters, and multiple inflammatory factors, are thought to be active in the tumor progression, but their roles vary.

### 3.1. Acetylcholine

Ach is a well-known neurotransmitter in the cholinergic system that is synthesized by choline acetyltransferase and acetyl-CoA. Various cells have been proven to have the ability to synthesize Ach, and tumor cells are one of them. Furthermore, studies have proven that the vagus nerve can suppress tumor cells [22]. Here, we mainly discuss Ach release from neurons. We have suggested that ablation of sensory neurons slows the initiation and progression of pancreatic cancer, and it has been found that the inhibition of cholinergic signaling and muscarinic receptors is important to suppress gastric tumorigenesis [3, 23]. Enhancement of the muscarinic signaling pathway in pancreatic cancer has been shown to directly inhibit tumor stem cells, CD11b+myeloid cells, TNF*α* levels, and hepatic metastatic growth through CHRM1 [24]. In 2013, NIE confirmed that acetylcholine (Ach) promotes the migration and invasion of hepatocellular carcinoma cells (HCC) and inhibits apoptosis by binding to androgen receptors [25]. Surprisingly, cancer cells seem to be able to upregulate their ability to grow or invade through the exploitation of locoregional neural plasticity. Moreover, there is enough evidence to support that the absence of splenic denervation or spleen-originated anti-inflammatory peptide TFF2 disrupts the anti-inflammatory nerve arc, triggering the expansion of myeloid-derived suppressor cells (MDSCs) and colorectal tumor development [26]. Thus, we confirmed the inhibitory effect of the vagus nerve on colorectal tumorigenesis achieved through its anti-inflammatory characteristics. In addition, it is of great importance to identify the relationship between the type of tumor and the source of innervation, which still needs more investigation. Two classes of Ach receptors have been identified: nicotinic acetylcholine receptors (nAchRs) and muscarinic receptors (mAchRs).

#### 3.1.1. nAchRs

nAchRs, also named nicotinic acetylcholine receptors, are expressed in both the PNS and central nervous system (CNS). At the CNS level, cholinergic signaling regulates stress and anxiety and induces mood-related behaviors, which is preclinical to cancer. However, because nicotine itself even facilitates tumorigenesis, it is difficult to determine how nicotine promotes tumorigenesis.

The activation of nAchRs promotes direct Ca^2+^ influx and accelerates many aspects of tumorigenesis, including cell proliferation, differentiation, epithelial-mesenchymal transition (EMT), migration, and invasion [27, 28]. The exposion to nicotine (nACH agonist) and carcinogens related to nicotine (NNN or NNK) can lead to the activation of pancreatic cells, resulting in the presentation of stem cell characteristics through the expression of CHRNA7 (cholinergic receptor nicotinic alpha 7 subunit) signaling and FOS-like 1 and AP-1 transcription factor subunit (FOSL1) activation of RNA polymerase II-associated factor (PAF1). Increased levels of PAF1 have been found in pancreatic tumors in humans and mice exposed to long periods of cigarette smoke [29]. Many studies have also shown that activation of nAchRs triggers interactions with other neurotransmitter receptors, which in turn activate various cascades. However, there is still no definite evidence that this mechanism participates in gastrointestinal tumors.

#### 3.1.2. mAchRs

A certain study demonstrated that the inhibitory effect of Ach plays a role in gastric cancer. Dclk1+ tuft cells, MKN45 and BGC823 gastric cancer cells, and Ach synthesized by nerve fibers were proven to activate M3R to facilitate tumor progression by stimulating the EGFR [30] and Wnt and YAP signaling pathways [31]. Meanwhile, the Wnt signaling and stem cell expansion were found in M3R inhibitor-treated or M3R gene-free models of gastric tumors. Activated M3R increases the invasion and metastasis ability of cancer cells by elevating the expression of MMPs in colon tumors.

Other researchers also verified that the Wnt signaling in stem cells mediated by vagus nerve regulation of M3R promotes the development of gastric cancer [32]. As previous studies have shown, M3R not only participates in gastric cancer but also participates in colon cancer and pancreatic cancer. In colon cancer, activated M3R upregulates MMPs to enhance the invasiveness and transfer ability of cancer cells [32]. In pancreatic cancer, a muscarinic agonist was found to suppress the generation of pancreatic tumors by blocking both the MAPK and PI3K/AKT signaling pathways [24]. Generally, M3R participates in a series of gastrointestinal tumors, which suggests that it is a potential therapeutic target in the future.

Ach is profoundly involved in the modulation of the inflammatory response [33], which regulates tumor development by altering the TME. Multiple immune cells express nAchRs and mAchRs and are activated by Ach [33].

Levels of TNF*α* in the spleen and circulation were suppressed by bethanechol in a mouse model of pancreatic cancer. Reduced CD11b+ myeloid cells were also confirmed in pancreatic tissues. This may be associated with tumorigenesis, as we mentioned above, suggesting that enhanced cholinergic signaling is a potential treatment target for the antitumor immune microenvironment in pancreatic cancer [24]. These studies suggested that Ach participates in the development of gastrointestinal tumors via the neuroimmune pathway.

### 3.2. Epinephrine (E) and Norepinephrine (NE)

As mentioned above, stress and chronic depression are risk factors for cancer. Multiple elements participate in these risks on the function of stress and depression of E and NE.

Epinephrine and norepinephrine, which are essential components of the fight-or-flight response, are profoundly involved in modulating the microenvironment of tumor tissues by taking part in various biological profiles of tumor cells, such as cell survival, proliferation, antiapoptosis, migration, angiogenesis, and matrix alternation [10, 34, 35]. NE can stimulate endothelial cell metabolism toward the inhibition of oxidative phosphorylation and the induction of an angiogenic switch that fuels cancer progression. People have shown that adrenergic signals promote the angiogenic switch in prostate cancer and that their inhibition could suppress the tumor growth, and the use of *β*-blockers therapy for the treatment of gastric tumors is well established [36, 37].

*β*-ARs are expressed in almost all immune cells and tumor cells [38]. In pancreatic cancer, NE secretion is regulated by positive feedback of catecholamines (including E and NE) that promote the synthesis of neurotrophins depending on *β*-AR. The elevated neurotrophins further increase the production of NE. This positive feedback loop facilitates tumor growth [10]. Monoamine oxidase A (MAOA), an important NE/E catabolic enzyme, is significantly decreased in HCC, diminishing HCC metastasis depending on NE inhibition of *β*-AR signaling and EGFR transactivation [39]. This process also increases the level of catecholamines in the tumor microenvironment. Furthermore, increasing E and NE promote tumors via several mechanisms, but they are largely mediated by *β*-AR-dependent increases in cAMP levels and subsequent activation of PKA. PKA executes relevant functional regulation by phosphorylating downstream targets [40]. Considering the analysis mentioned above, studies on *β*-AR and PKA still have great potential.

Similar to Ach, NE and E can also influence the TME indirectly. *β*-AR, as mentioned above, is an inevitable topic. If tumors can successfully escape immune attack, they can develop “normally.” Humans establish an immune surveillance system, such as a great wall, with multiple kinds of immune cells. *β*-AR-mediated hormone signaling reduces the deformability of macrophages [41] and modulates integrin activation in human antigen-specific T cells [42]. For primary tumors, *β*-AR stimulation is enough to elevate the invasion of CD11b (+) F4/80(+) macrophages into their parenchymal tissues and trigger a signal expressing a premetastatic gene together with an indication of M2 macrophage differentiation [43]. Inhibiting the *β*-AR signaling pathway or chemically eliminating the sympathetic nerves effectively eliminates the effect of EEs on NK cells and weakens the antineoplastic function of EEs [17]. Therefore, the significant role of *β*-AR in the regulation of the immune system makes it a new therapeutic target for cancer. Many studies have suggested that *β*-blockers have a remarkable reduction in the prevention and treatment of the breast and prostate cancer [44, 45]. However, some clinical research verified that no beneficial effects of *β*-blockers were present in patients with colorectal cancer, while in patients with pancreatic cancer and prostate cancer, *β*-blockers showed certain untoward effects on overall survival [46], which contradicted the studies above.

### 3.3. Gamma-Aminobutyric Acid (GABA)

**Figure 1:**
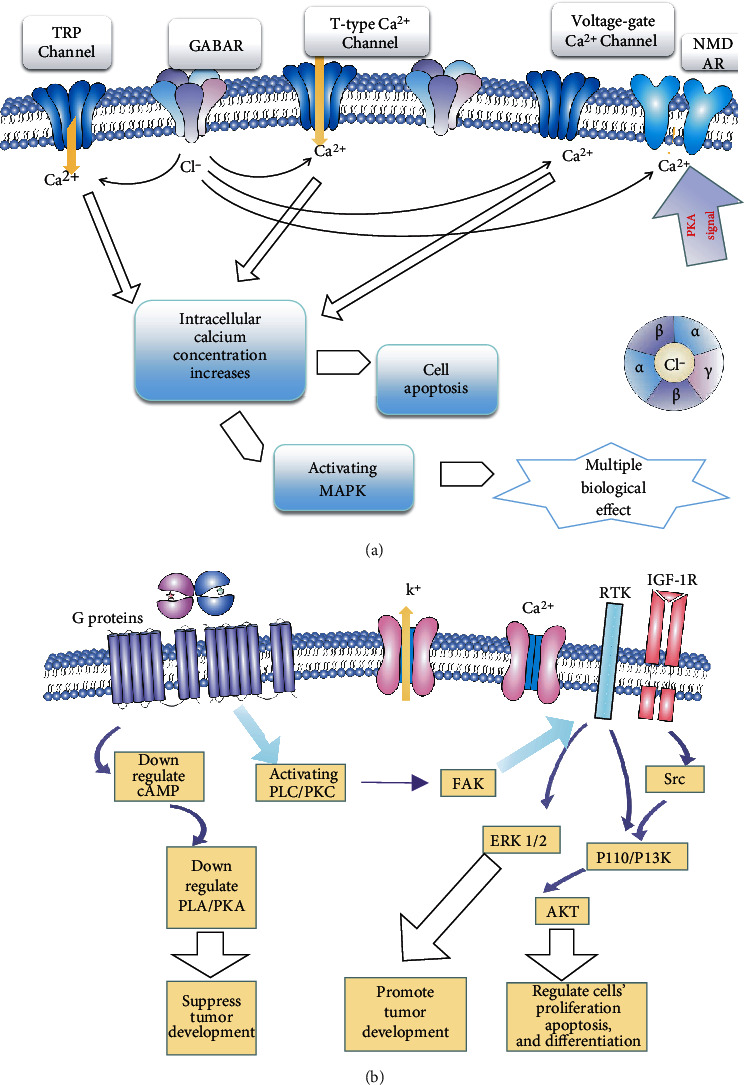
The functions and molecular mechanism of GABA and its receptors in cancer development. (a) The GABARs contain 5 major subunits, and stimulation of GABAR with GABA will activate the chloride ion channel. The change of electric potential further activates the calcium ion channel and increases the intracellular calcium concentration. The activation of the PKA pathway also increases calcium concentration. The higher concentration of calcium promotes tumor cell apoptosis and activates the MAPK pathway to achieve multiple biological effects. (b) The GABBRs are G protein-coupled receptors. When agonists are combined, it will suppress the cAMP pathway and promote the PKC pathway. PKA suppression inhibits the growth of tumor, while the activation of the PLC/PKC pathway regulates multiple activities via FAK which are associated with IGF1R and RTK. The activation of PKC increases intracellular calcium concentration to induce cell apoptosis. Additionally, other signaling pathways such as YAP also participate in the process of GAGBR-dependent tumor-suppressive or oncogenic roles.

GABAergic signaling is involved in immune inflammatory disorders and the function of immune cells. GABAergic signaling potentially participates in the immune network in various inflammatory disorders and has a significant impact on the multifaceted function of immune cells. For example, multiplication of antigen-induced T cell cytokines induced by LPS activates cytotoxicity and induces chemotaxis of effector T cells [47, 48]. On the other hand, GABA also regulates the cytotoxicity of immune-active cells, which possess GABAA receptor subunits [49]. Interestingly, the existence of GABA in tumor tissues implies that it might participate in inflammatory reactions by anchoring invasive immune cells [50]. Moreover, recent studies show that GABA secreted from B cells provokes a protumor immune environment [51, 52]. GABA is mainly derived from gastric cancer cells [53–57] and participates in tumorigenesis via PNI. There are two kinds of GABA receptors that we discussed, including GABAA receptors and GABA_B_ receptors. Up to 19 different GABAAR subunits (*α*1–6, *β*1–3, *γ*1–3, *δ*, *ε*, *θ*, *π*, and *ρ*1–3) have been identified in GABAR [58], and their functions vary. The GABAA receptor is upregulated in pancreatic cancer [50, 59, 60] but downregulated in liver cancer and pancreatic cancer [61, 62]. In GABAR, different subunits have different functions. Recently, a study showed that the *π* subunit of the GABAA receptor promotes pancreatic cancer progression by tuning KCNN4-mediated Ca^2+^ in a GABA-independent manner [50]. In HCC, GABAR-*β*3 is expressed as an inhibitory factor [63], while its *α*3 subunit promotes HCC cells [64]. Muscimol is a GABAA receptor stimulant that enhances tumor cell growth in gastric organs by activating MAPK. Likewise, GABA raises extracellular Ca^2+^ levels and upregulates the MAPK/ERK signaling cascade by elevating GABRP (a subunit of GABAA) expression, resulting in stimulation of pancreatic cancer development [65]. Conversely, GABAB receptor activity potently suppresses cAMP-associated stuffs, such as isoproterenol-induced cAMP, cAMP response element luciferase, p-CREB, and ERK1/2 phosphorylation, thus efficiently preventing DNA synthesis and cell movement [66]. Diverse influences of activated GABA on tumor development/movement might depend on the type of GABA receptor or specification of the cancer cell [50]. The GABAA receptor mediates the enhancement effect on the proliferation of tumor cells, while the GABAB receptor contributes to the inhibition effect on tumor cell growth [67] (Figure 1).

Jiang et al. revealed that GABRP modulates the recruiting function of macrophages in pancreatic tumor tissues by increasing the levels of CXCL5 and CCL20 [50]. However, the precise mechanisms of how GABA and its signaling network mediate the immune cell and cancer microenvironment remain to be explored [11]. GABA is utilized to lower blood pressure, maintain one's composition, and lower blood glucose levels [68]. In addition to the medicinal effect of GABA, certain GABA receptors are also used for sedation in addiction [69]. At the same time, research has demonstrated that benzodiazepine, as a sedative that depends on the activation of GABA, increases the risk of liver, gastrointestinal, and pancreatic tumors in a dose-dependent manner but has no similar effect on ovarian tumors, malignant melanoma, or colon tumors [20]. Accordingly, GABAergic agents are a valuable target medicine for further exploration [50]. Here, we list some studies that discuss GABAergic agents and GABA-related medicines targeting tumor treatment.

**Figure 2:**
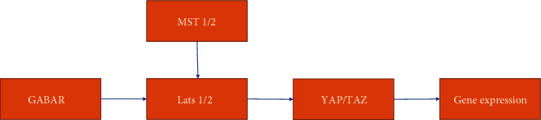
The YAP/TAZ pathway and tumor gene proliferation. The Hippo signaling pathway, also known as the Salvador/Warts/Hippo (SWH) pathway, is named after Hippo (Hpo), a protein kinase in drosophila and a key regulatory factor in the pathway. This pathway is composed of a series of conserved kinases that control organ size by regulating cell proliferation and apoptosis. Once it senses the extracellular growth inhibition signal, it activates a cascade of kinase phosphorylation reactions, culminating in phosphorylation of downstream effector factors YAP and TAZ. Cytoskeleton proteins bind to phosphorylated YAP and TAZ, causing them to remain in the cytoplasm and reduce their nuclear activity, thus achieving regulation of organ size and volume.

According to the conclusion mentioned above, people are focusing on GABA-related treatment from two different perspectives. Some researchers have focused on GABAB receptors, and others have focused on GABAA receptors. More studies now put GABBR at a significant place in tumor treatment. A study suggests that GABAB receptors regulate proliferation in the high-grade chondrosarcoma cell line OUMS-27 via apoptotic pathways [70] and offers clinical treatment from a new perspective. Additionally, the inhibition of the receptor, an opposite measure, is used in prostate cancer aimed at EGFR lines [71]. In CRC, GABBR1-related research unveils a Hippo/YAP1 signaling pathway to demonstrate that GABBR1 inhibits the development of CRC and indirectly shows the feasibility of treatment targeting GABBR1 [72] (Figure 2). A study pointed out the prospects of GABAA receptors [73], and this article listed the complex internal connection of GABAA receptors and different organs. Kleinerman et al. conducted a retrospective analysis 38 years ago and reported that the use of benzodiazepine diazepam reduced primary tumor size and reduced the incidence of lymph node involvement in breast cancer patients [74]; however, we discussed above that mood function is connected to tumorigenesis and that GABA could decrease the anxiety of patients. This analysis failed to distinguish the true reason for this consequence. GABAR's ability to mediate the influx of calcium ions promotes apoptosis. Strikingly, studies have indicated that benzodiazepine has a depressive effect in melanoma and brain cancer medulloblastoma. A series of benzodiazepine analogs that had a preference to bind to GABRA5 containing GABAAR impaired the viability of cells in culture (IC50 1–0.1 micromolar) and induced apoptotic responses in vivo, and the effect in vivo was more significant and specific than that of standard-of-care chemotherapeutics [75, 76]. These studies point out that GABA-related treatment is a welcomed weapon to anticancer therapy. Although there are only a few clinical studies to inform GABAergic agents and GABA antibodies contributing to the treatment of digestive tumors, a great amount of evidence suggests that studies on this issue still have great potential. There are still many problems waiting to be addressed, such as how GABAR may mediate crosstalk in the tumor microenvironment between noncancer cells, including immune cells and vessel cells. Furthermore, the structure and function of GABAR subunits and GAGBR subunits also need to be fully discovered in future clinical studies.

### 3.4. Serotonin

5-Hydroxytryptamine (5-HT) is a neurotransmitter, and more than 90% of it is produced by chromaffin cells in the intestine and then stored in platelets. The remaining 5-HT is synthesized by serotonergic neurons in the brain. 5-HT acts as an important modulator of human behaviors, including memory, mood, sleep, appetite, and temperature [50]. 5-HT is thought to maintain epithelial homeostasis in the pancreas and liver tissues. This might explain the high frequency of imbalanced 5-HT signaling in epithelial cancers [77, 78]. In liver cancer, 5-HT promotes tumor growth by inhibiting autophagy, and inhibition of 5-HT signaling by targeting the 5-HT2B receptor consistently impairs tumor growth. In hepatic tumors, 5-HT enhances the development of tumors by blocking autophagy and suppressing 5-HT signaling by anchoring the 5-HT2B receptor to prevent tumor development persistently [79]. In PDAC, 5-HT synthesized by tumor tissue enriches the TME. It should be noted that the 5-HT receptor 2B levels increase in PDAC cells, increasing the glucose catabolism of tumor tissues when facing metabolic stress and then enhancing the progression of PDAC [80]. Pancreatic cancer cells can absorb 5-HT by transport-mediated means. Large and rapid intracellular accumulation of 5-HT greatly activates the small GTPase Ras-associated C3 botulinum toxin substrate 1 (RAC1), which is an important process during acinar-to-ductal metaplasia (ADM) [81].

In addition, platelet-originated 5-HT also accelerates the development of tumor tissue angiogenesis and cancer cell invasion ability [82]. Notably, tumor angiogenesis was prevented by suppression of endothelial NO synthase and P-ERK1/2 causing 5-HT depletion and selective inhibition of 5-HT2B receptors [83]. All the above findings suggest that the 5-HT signaling pathway critically participates in the neogenesis and progression of cancer. 5-HT is a multifunctional molecule that regulates the immune system [84]. It deals with multiple immune activities, from chemotaxis to leukocyte activation, proliferation, and cytokine secretion. All of these target cell-specific activities depend on membrane receptors such as SERT and 5-HTR that regulate the response to 5-HT downstream of the signal transduction pathway; 5-HT metabolic enzymes, such as indoleamine 2,3-dioxygenase 1 and monoamine oxidase, produce bioactive catabolites such as kynurenines and kynurenine. Unfortunately, the effect of the 5-HT system in the microenvironment of cancer tissues has not been well studied. Many 5-HT therapies have been harnessed to treat gastrointestinal tumors, especially to treat some chemotherapy complications [85]. A recent study verified that SB204741, a specific antagonist of HTR2B, significantly inhibited cancer cell growth in pancreatic tissue by blocking Warburg function [80].

### 3.5. Neuropeptides

Neurons continue to communicate with each other by small protein-like molecules called neuropeptides. In gastrointestinal tumors, current studies are focusing on the SP/NK1 system. Once activated, the neurokinin-1 (NK-1) receptor, coupled to the Gq family of G proteins, synthesizes two second messengers (inositol 1,4,5-triphosphate (IP3) and DAG) and mediates the biological action of SP [86]. However, malfunction of the SP/NK-1 system often occurs regardless of whether it participates extensively in the neogenesis and progression of digestive tumors (colon, pancreatic, and gastric tumors) [86, 87]. Pharmacological inhibition of the NK-1 receptor with specific antagonists (aprepitant, fosaprepitant, L-732,138, and L-733,060) results in pronounced antitumor effects [88, 89]. Investigations aimed at neuropeptides are still in progress, and more attention is needed in the future.

### 3.6. Dopamine

It seems that dopamine and its receptor (DR) agonists present antitumor functions in gastric cancer [90] but show no effect on the growth and migration of colon cancer cells [91]. The different features might be explained by separate tumor types, DR levels, and dosages. Some feeble clues suggest the reason may be due to the different tumor types. They found that suppression of DRD2 decreases the growth and invasion of pancreatic cancer cells, thus slowing down the growth of xenograft tumors in mice [92].

Dopamine takes part in inhibiting tumor neogenesis in the adrenergic system, but whether the *β*-AR signaling is involved in dopamine prevention is under exploration [11] (Table 1).

## 4. Neuroendocrine-Immune Mechanism in Gastrointestinal Tumor

Tumorigenesis is a systematic process. The promoting or inhibiting effects of nerve cells are closely related to the NEI system. As mentioned above, external psychosocial processes activate cortical and limbic structures of the central nervous system, and subsequent effects stimulate the hypothalamic–pituitary–adrenal (HPA) axis and sympathetic nervous system (SNS), activating defeat/withdrawal responses and fight-or-flight stress responses, respectively [93]. On the HPA axis, the inducing effect of nerve fibers promotes the release of a series of hormones, such as CRH, secreted by the paraventricular nucleus of the hypothalamus, stimulates the anterior pituitary to produce adrenocorticotrophic hormone, and then induces the secretion of glucocorticoid hormone cortisol by the adrenal cortex. SNS activation enhances the release of NE and E. Evidence suggests a strong correlation between changes in neuroendocrine dynamics and tumor pathogenesis [94], and it is apparent that nerves are bound to this process. Regarding immunity, neuroendocrine factors, especially catecholamines and cortisol, were proven to be able to modulate the immune system related to cancer surveillance [95]. The role of catecholamines has been described above, while cortisol also has a complex immune and endocrine effects on gastrointestinal cancer.

Recent studies demonstrated that a sharp rise in stress-triggered glucocorticoid levels and increased Tsc22d3 levels in DCs might change chemotherapy- or immunotherapy-induced antitumor immune surveillance. A close correlation was found between plasma cortisol levels and TSC22D3 levels in leukocytes and negative moods in a group of cancer patients [12]. In addition, the endocrine arrhythmicity induced by stress or day-night imbalance is widely accepted to increase the risk of tumorigenesis [93], and it might be due to the disorder of various hormonal, neurological, and immune factors.

Prevailing concepts are gradually recognizing that obesity is involved in a range of neuropsychological disorders that are influenced by a variety of factors, including psychosocial factors related to stress and addictive behaviors, rather than just endocrine disorders [96, 97]. Secondary hyperleptin also increases the risk of cancer, in addition to the promoting effect on disturbance of the autonomic nervous system, resulting in the creation of a facilitating environment to enhance tumor development. The mechanism by which obesity is conducive to tumorigenesis has been mentioned above. On the other hand, both the disturbance of endocrine hormones and disorders concerning nervous centralis combined with cachexia-associated transmitters can also lead to katabolism in fat tissue [98].

Epidemiological studies of circadian imbalance present strong relevance to hormone-related cancers (such as breast cancer), but it remains to be investigated in cancers of the digestive system [99]. Recent findings have explained how tissues coordinate themselves and communicate with each other within the whole system by biological clocks [100, 101]. The suprachiasmatic nucleus, located in the hypothalamus, adjusts biological rhythms through neuroendocrine modulation to keep all of the organs synchronized [102]. Based on this theory, cancer cells can disturb the systemic circadian clock to provoke many processes, including multiorgan chronic inflammation, metabolic disorders, and cachexia, by secreting related hormones and cytokines [98]. A remarkable paper suggested that lung adenocarcinoma distally rewires circadian transcription and metabolism by altering proinflammatory responses via the secretion of IL-6, TNF-*α*, and lactate [103]. However, in this case, it remains a mystery whether the metabolic disequilibrium of hepatocytes further facilitates tumors. Subsequently, a preclinical study revealed an interconnection between day-night rhythm and tumor growth at the molecular level. Disturbance of circadian rhythms promotes disruption of the peripheral clock of cholestasis and sympathetic dysfunction, leading to activation of a remarkable cancer cell promoter in the liver, constitutive androstane receptor, contributing to the development of liver cancer induced by nonalcoholic fatty liver disease (NAFLD) [104].

Basically, neurohormone and immune factors are mutually fueled. Exposure to various stresses is found to initiate the cascade progress. The first step is the disturbance of purine metabolism in CD4-positive T cells and agitated behaviors [105].

Meanwhile, metabolic changes in the transmitter of nerves, as well as disturbance in the secretion level of neurohormones, occurred. In addition to these changes, neuroplasticity is ultimately affected by innate immune cells through the secretion of various cytokines, suggesting a strong correlation between the NEI axis and tumorigenesis [106, 107]. These mechanisms suggest a strong connection between the NEI axis and tumorigenesis. Moreover, depression can aggravate cytokine responses to pathogens or stressors and exert multiple effects on tumorigenesis. In summary, chronic stress and long-term and unrestricted inflammation triggered by obesity and sleep disturbances can further promote tumorigenesis [108].

## 5. Conclusion

From what has been discussed above, we reviewed the correlation between nerves and gastrointestinal tumors. This association involves a variety of regulatory mechanisms, including central-level regulation and PNI and NEI regulation. Transmitter and nerve ending play a crucial role in all aspects. At the central nervous system level, emotional behavior plays significant roles in the development of cancer. In peripheral nerves, PNI is an essential step of cancer development. The NEI axis and neurotransmitters play their own roles in tumorigenesis.

Finally, we suggest that medical or surgical therapies aimed at treating gastrointestinal tumors in nerves need further study, thus providing a new perspective on the management of digestive cancers.

## Figures and Tables

**Table 1:** A comparison between serotonin, neuropeptides, and dopamine.

	Related functions	Regions	Probable effect on tumor development		Refs.
			Promoting factors	Inhibiting factors	
Serotonin	(1) Regulation of liver and pancreas epithelial homeostasis (2) Modulation of immune system	Liver, pancreas	(1) Suppression of autophagy (liver cancer) (2) Enriching TME and increasing glycolysis (PDAC) (3) Promotion of tumor angiogenesis	Unknown	[77–80, 82–84]
Neuropeptides	Medium of communication between neurons	Colon, pancreas, gastric	Dysregulation of SP/NK1 system	Unknown	[86–89]
Dopamine	Waiting for further studies (might be relative to concrete tumor type)^∗^	Pancreas, gastric	Unknown	Antagonizing adrenergic system (waiting further investigations)^∗^	[11, 90–92]

^*^Promising investigation target in authors' view.
